# An improved method for the *in vitro *differentiation of *Plasmodium falciparum *gametocytes into ookinetes

**DOI:** 10.1186/1475-2875-9-194

**Published:** 2010-07-08

**Authors:** Anil K Ghosh, Rhoel R Dinglasan, Hiromi Ikadai, Marcelo Jacobs-Lorena

**Affiliations:** 1 Johns Hopkins University School of Public Health, Dept. of Molecular Microbiology and Immunology, Malaria Research Institute, 615 N. Wolfe St., Baltimore, MD 21205, USA; 2 Department of Veterinary Parasitology, School of Veterinary Medicine, Kitasato University, Towada, Aomori 034-8628, Japan

## Abstract

**Background:**

Ookinete is the form of the malaria parasite that invades the mosquito midgut epithelium to initiate sporogony. Differentiation of ingested gametocytes into ookinetes in the mosquito midgut lumen and subsequent interaction with the lumenal surface of the midgut epithelium in preparation for invasion is a complex and multi-stepped process. To facilitate the study of these events in detail it is necessary to produce sufficient numbers of pure, fully mature and functional ookinetes. However, production of even a small number of *Plasmodium falciparum *ookinetes *in vitro *has proven to be a daunting task. Consequently, over the past four decades our collective understanding of the biology of this parasite form remains sorely deficient. This article reports on investigations of five different ookinete media, in an effort to improve the *in vitro *transformation efficiency of *P. falciparum *gametocytes into mature ookinetes and their infectivity of the mosquito midgut.

**Methods:**

Five different ookinete media were evaluated for their ability to support the differentiation of gametocytes into gametes and further into mature stage V ookinetes. Moreover, infectivity of the *in vitro*-transformed ookinetes was evaluated by feeding them to vector mosquitoes and measuring their ability to traverse the midgut and form oocysts.

**Results:**

One of the five media (medium E) was clearly superior in that the cultured ookinetes produced the largest number of oocysts when fed to mosquitoes. Key components were additions of human serum, human red blood cell lysate and mosquito pupal extract, resulting in the production of larger numbers of ookinetes able to develop into oocysts when fed to mosquitoes.

**Conclusion:**

This simple and practical improvement over the prevailing methodology will facilitate the investigation of how this important human malaria parasite initiates its development in the mosquito and will contribute to the understanding of its transmission biology.

## Background

*Plasmodium*, the causative agent of malaria, infects an estimated 500 million people every year and has the highest health impact on women and young children in sub-Saharan Africa [1]. Parasite resistance to available drugs and vector mosquito resistance to insecticides have hampered the fight of this devastating disease. Moreover, despite massive efforts, an effective vaccine has not yet been developed. New strategies need to be developed. One approach is to interrupt parasite transmission by the mosquito vector, an approach that requires in depth understanding of parasite development in the mosquito.

Soon after the mosquito ingests an infected blood meal, gametocytes differentiate into gametes that mate to form zygotes and later motile ookinetes. To exit the mosquito midgut lumen, ookinetes traverse the midgut epithelium and lodge beneath the basal lamina where they differentiate into oocysts. Upon maturation, each oocyst releases several thousand sporozoites into the haemocoel from where they invade the salivary glands. At this point, the sporozoites are ready to be transmitted when the mosquito takes a blood meal from another vertebrate host [2]. Little is known about the developmental processes that operate during the differentiation of gametocytes into ookinetes [3].

While gametocytes can be easily obtained from an *in vitro Plasmodium falciparum *culture, current methods for the transformation of gametocytes into ookinetes are poor with a reported transformation efficiency of only 0.002% (0.2 mature ookinetes per 10,000 red blood cells (RBCs)) [4]. Moreover, the ability of these ookinetes to develop into oocysts in the mosquito has not been determined [4]. This is in contrast with the *in vitro *differentiation of the rodent parasite *Plasmodium berghei*, which is efficient and yields about 10^6 ^ookinetes from a single infected mouse [5]. The lack of an efficient *P. falciparum *differentiation protocol has hampered the study of ookinete differentiation and its interactions with the mosquito vector.

In the work presented here, a culture medium was established that supports the efficient development and differentiation into mature *P. falciparum *ookinetes. The medium described by Carter *et al *[4] was modified by replacing 20% foetal bovine serum (FBS) with O-positive human serum (medium A). Four additional media (media B-E) were created by addition of various supplements and functional integrity of the resulting ookinetes was verified by measuring their infectivity to mosquitoes.

## Methods

### Materials

RPMI 1640 (Invitrogen), Schneiders medium (Invitrogen), Waymouth medium (Invitrogen), O-positive human serum (Interstate Blood Bank), xanthurenic acid (Sigma), hypoxanthine (Sigma), anti-Pfs25 mouse monoclonal antibody (obtained from the Malaria Research and Reference Reagent Resource Center (MR4)), mouse monoclonal anti-*P. falciparum *chitinase antibody (a gift from Dr. Joseph Vinetz), goat anti-mouse IgG labeled with Texas Red (Invitrogen), antibody labeling Kit (Pierce).

### Mosquitoes

The colony of *Anopheles gambiae *(Keele strain) was obtained from Drs. Hilary Hurd and Paul Eggleston at Keele University. Larvae were reared on dry cat food. Adults were maintained on 10% sucrose solution at 27 ± 1°C and 80 ± 5% relative humidity with a 14 h/10 h light/dark cycle. About 5- to 6-day-old *An. gambiae *mosquitoes were starved overnight and selected by placing a bottle with water at 37~40°C next to the cage (touching a meshed side of a cage or the side of an ice cream container). Host-seeking mosquitoes that are attracted to the heat were transferred by aspiration to separate 16 oz. feeder cups.

### Parasite culture

*Plasmodium falciparum *NF54 parasites (0.5% parasitemia) were maintained at 5% haematocrit in *P. falciparum *culture medium (RPMI 1640 medium containing L-glutamine supplemented with 0.2% sodium bicarbonate and 10% v/v O-positive human serum) in a 37°C chamber with 'malaria gas mixture' (5% CO_2_, 5% O_2 _and 90% nitrogen) [6,7].

### Gametocyte cultures

Gametocytogenesis was induced by stopping addition of human RBCs and by changing 'the medium daily until day 18. On alternate days about 10 μl of the culture was smeared on a slide, fixed with methanol and stained in Giemsa stain (1:40 dilution) to count the gametocytes and the male:female ratio.

### Preparation of *An. gambiae *pupal extract

About 500 *An. gambiae *pupae were collected, washed with sterile distilled water several times and after a final wash in PBS they were stored at -80°C until use. Frozen pupae were thawed, resuspended in 500 μl of PBS and transferred to a glass homogenizer. Homogenization was done on ice with 10 to 12 strokes and the homogenate was centrifuged at 14,500 *g *for 10 min at 4°C. The clarified supernatant was transferred to a clean tube, heated at 60°C for 1 h and after cooling to room temperature it was centrifuged at 14,500 *g *for 30 min at 4°C. The clarified supernatant was aliquoted and stored at -80°C until use.

### Preparation of human red blood cell (RBC) lysate

O-positive human RBCs were washed (about 0.5 ml of packed volume) in sterile PBS. RBCs were lysed by adding 900 μl of sterile distilled water and swirled for 30 sec for lysis to occur. Immediately following lysis, 100 μl of 10× PBS was added to the tube to make it isotonic. The lysate was centrifuged at 14,500 g for 30 min at 4°C. The supernatant was aliquoted and stored at -80°C until use.

### Preparation of ookinete media

All components of the medium were mixed, the pH was adjusted and then sterilized by passage through a Corning 0.2-micron filter sterilization unit. Table 1 provides the composition of the five media tested in this study. To prepare 100 ml of final ookinete differentiation medium (E) 22.6 ml RPMI 1640, 22.6 ml Schneiders medium, 22.6 ml Waymouth medium, 20 ml human serum, 1 ml 50 μM hypoxanthine, 5 ml 4% sodium bicarbonate, 2 ml pupal extract, 4 ml human RBC lysate were added and the pH was adjusted to 7.4 with 10N NaOH.

**Table 1:** Composition of five ookinete differentiation media

**Medium**^**a**^	Components
A	RPMI 1640, 20% Human group A serum, Hypoxanthine (50 μg/ml), Sodium bicarbonateb (2 g/l).
B	RPMI1640 and Waymouth medium (1:1) Remaining components are the same.
C	RPMI 1640, Waymouth, and Schneider's Drosophila Medium (1:1:1). Remaining components are the same.
D	Same as medium C with 0.2 μM xanthurenic acid.
E	Same as medium C with pupal extract and human blood lysate.

^a ^The pH of all media was adjusted with 10 N NaOH every time before use.

^b ^The sodium bicarbonate solution was prepared freshly at the time of medium preparation.

### Counting exflagellation centers

About 100 μl of the gametocyte culture was sampled on day 18 and centrifuged at 650 *g *for 5 min followed by resuspension of the packed cells in the same volume of ookinete medium. A drop (4-6 μl) of the resuspended gametocytes was placed on a slide. After breathing on a cover slip, it was placed over the droplet, followed by incubation at room temperature (22°C) to induce exflagellation. After 10 min the slides were observed with differential interference contrast (DIC) optics to count exflagellation events at five positions of the cover slip (4 corners and the center of the cover slip, all together 25 fields; the average value was used) using a 40× objective. If no exflagellation events were observed at 10 min, slides were checked every 2 min until exflagellation was observed.

### *In vitro *differentiation into ookinetes

After checking gametocytaemia, about 3 ml of the gametocyte culture was transferred into a conical 15 ml COSTAR tube and centrifuged at 650 *g *for 5 min at 37°C. The cell pellet was washed three times with incomplete medium (RPMI 1640 medium only, no serum). After the final wash, packed cells were resuspended in 250 μl of each of the five ookinete culture media (Table 1). Cells were gently resuspended in each medium, transferred into a well of a 24-well tissue culture plate and incubated at 24 ± 2°C on a shaker (50 revolutions per min) for 24 h.

### Evaluating stages of ookinete differentiation

About 100 μl of the ookinete culture was centrifuged at 650 *g *for 5 min and the supernatant was discarded. Packed cells were resuspended in 20 μl of human serum by flicking the tube and the suspended cells were used to make a thin smear on 2~4 slides, followed by methanol fixation for 3 min at room temperature. Slides were air-dried and stored at room temperature for subsequent histological analysis after Giemsa staining. Different stages of ookinete differentiation were counted by microscopic examination of the Giemsa-stained slides using a 100× oil-immersion objective and images were recorded. Total number of each ookinete stage per 10,000 RBCs was determined. For immunostaining slides were fixed with 4% paraformaldehyde (Fisher Scientific) for 1 h at 4°C. The slides were washed with PBS and stored at -80°C for later use.

### Ookinete surface staining

Slides containing paraformaldehyde-fixed cells (see above) were thawed and air-dried at room temperature and blocked in blocking solution (PBS with 4% BSA, pH 7.4) for 1 h. Slides were incubated with anti-Pfs25 monoclonal antibody (diluted 1:1000 in blocking solution) for 1 h at room-temperature and washed several times at 5 min intervals with PBS. Slides were blocked in blocking solution as described above and incubated for 45 min at room temperature and in the dark with Texas Red-labeled anti-mouse IgG (1:2000 dilutions in blocking solution). Slides were washed several times with PBS, mounted in Slowfade ProLong Gold antifade reagent with DAPI (Invitrogen) mounting medium, overlaid with a coverslip and sealed with nail varnish. Slides were kept in the dark at 4°C until image acquisition.

### Double staining

Anti-Pfs25 antibodies were conjugated with FITC using the Pierce antibody labeling kit and following the manufacturer's protocol. Slides containing paraformaldehyde-fixed cells were thawed, air-dried and permeabilized with blocking buffer containing 0.1% Triton X-100 (Sigma) for 1 h at room temperature. Slides were incubated with anti-Pf chitinase mouse monoclonal antibody (1:200 dilutions) for 1 h at room temperature. Slides were washed in PBS and blocked again followed by incubation for 45 min at room temperature and in the dark with Texas Red-labeled anti-mouse IgG (1:2000 dilution in blocking solution). Slides were washed in PBS and blocked again followed by incubation with the FITC-labeled anti-Pfs25 monoclonal antibody (see above; 1:100 dilution in blocking solution). The slides were washed several times with PBS and mounted in Slowfade ProLong Gold antifade reagent with DAPI (Invitrogen). All images were acquired and processed equally with Photoshop, using the same parameters.

### Membrane feeding and measurement of oocyst numbers

Clean membrane glass feeders were connected with 4 inch-long tubing in a row (maximum six feeders) and the tubing was connected to the outlet manifold of an immersion circulating water bath that had been set to 40 ± 2°C. Just before adding cultures to the feeders, a piece of Parafilm (1 square inch) was taken and stretched across the open bell-shaped end of the feeder, to make a shallow feeding chamber. Ookinete culture was introduced into the chamber making sure that the culture covered the entire feeding surface. Each feeder was placed over a container with mosquitoes that had been starved overnight at 27 ± 1°C and 80 ± 5% relative humidity and mosquitoes were allowed to feed for 15 min.

#### Gametocyte feeding

Human blood (RBCs) was washed three times with human serum and resuspended to the original volume with human serum kept at 37°C. Gametocyte culture was centrifuged at 650 *g *for 5 min at 37°C and the supernatant was removed. The packed cell volume of the pellet was measured and washed RBCs were added using a ratio 1:3 (vol/vol), mixed well and the mixture was kept in a 37°C water bath until feeding.

#### Ookinete feeding

Mature ookinetes (24-hour culture) were centrifuged at 650 *g *for 5 min at room temperature. The packed cell volume of the pellet was measured and the pellet was resuspended with washed human RBCs (prepared as above) using a ratio 1:3 (vol/vol), mixed well and the suspension was kept at room temperature until feeding using membrane feeders as described earlier. Mosquitoes were allowed to feed for 15 min in the dark.

Unfed mosquitoes were removed at ~24 h after feeding and the fed mosquitoes were kept for 8 d with 10% sucrose solution in an incubator at 27 ± 1°C and 80 ± 5% humidity. On day 8 post-feeding mosquitoes were dissected and incubated with 0.2% mercurochrome (Sigma) in sterile distilled water; for 10-20 min. Midguts were washed in PBS and mounted on a slide with PBS. The number of stained oocysts in each gut was counted at 20× or 40× magnification.

### Microscopy

Images were captured using a CCD camera and Spot 3 software using a 100× oil immersion objective. Images were merged using Adobe Photoshop 7.

## Results and Discussion

### Development of gametocytes

**Figure 1:**
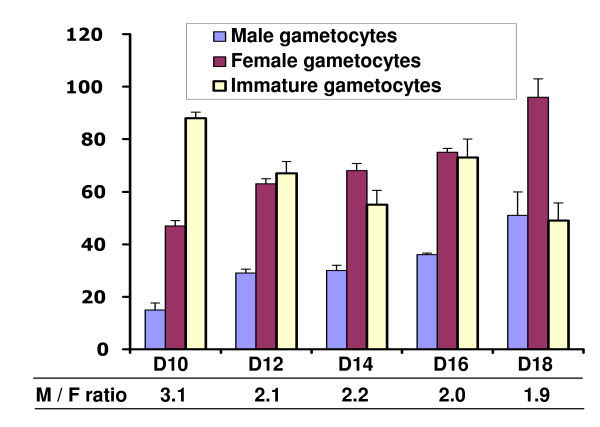
**Induction of gametocyte differentiation, exflagellation, and stages of ookinete differentiation**. The number of male, female and immature gametocytes per 10,000 RBCs was counted at different days after culture in gametocyte-induction medium. No gametocytes were observed before day 10. The male-to-female (M/F) ratio is indicated on the bottom line. The graph displays the mean of three independent experiments. Error bars represent standard deviation.

*Plasmodium falciparum *gametocyte cultures started to produce gametocytes on day 10, at which time the ratio of male to female gametocytes was about 3 (Figure 1). From day 12 onward mature male gametocyte production increased and the male to female ratio gradually changed to about 2. This is less than the 1:2.9 ratio that was observed in the field [8,9].

Several procedures have been employed to induce gametocyte formation, but the most reproducible and convenient one is to stop adding fresh RBCs while changing media daily. This was routinely used to obtain mature gametocytes on day 18. These cultures typically had 2~3% gametocytaemia and provided the starting material for the experiments reported below.

### Transformation of gametocytes to gametes

**Figure 2:**
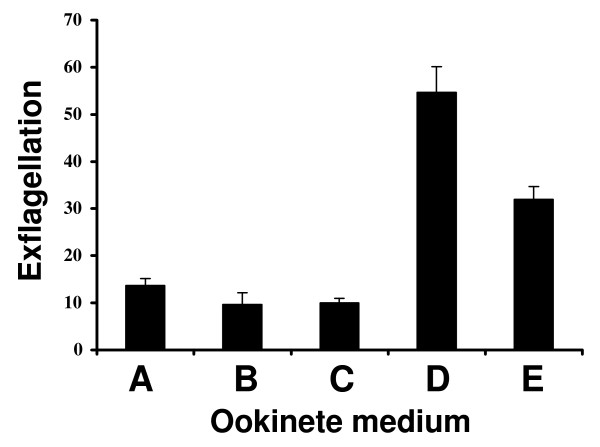
**Number of exflagellation events per field of male gametocytes after suspension in each of the five ookinete media (A-E; Table 1)**. For each experiment 25 fields were counted and the mean number of exflagellation centers per field was determined. The graph reports the results of three independent experiments. Error bars represent standard deviation.

Each of the media listed in Table 1 was assessed for its ability to promote differentiation of gametocytes into gametes. Day 18 gametocytes were washed three times in incomplete medium, transferred into the each of the five ookinete media and the number of exflagellation centers was counted. Media A-C yielded 10 to 15 exflagellation centers per field, while this value was 50~55 for medium D and 30~32 for medium E (Figure 2).

*In vitro *induction of exflagellation is normally accomplished by lowering of the temperature and addition of xanthurenic acid [10]. Media A-C contain only basic components and no specific gamete-inducing factors, therefore exflagellation was likely induced by the drop in temperature from 37°C to 22°C. The high exflagellation values achieved by medium D was likely due to the added xanthurenic acid [11] and for medium E due to the presence of xanthurenic acid in the pupal extract [10].

### Morphology of ookinete stages

**Figure 3:**
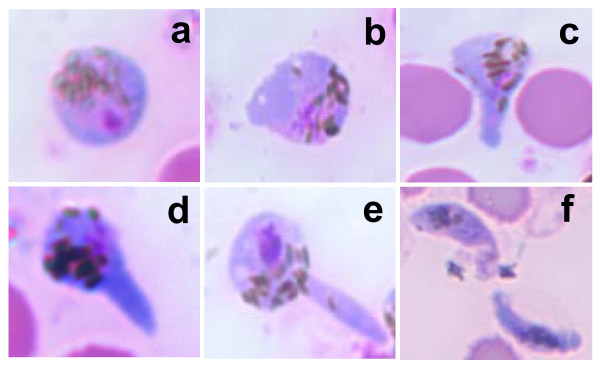
**Stages of ookinete differentiation**. **a: **zygote after 6 h of culture. **b-f: **stages 1 to 5, respectively. Differentiation was performed in medium E.

The morphology of the different stages of ookinete development was assessed microscopically with Giemsa-stained smears (Figure 3). The zygote forms within 6-8 h, has a laterally displaced round-shaped nucleus and displays distinct haemozoin pigment (Figure 3a). The stage 1 ookinete has a small protrusion and contains haemozoin pigment (Figure 3b). The protrusion continues to elongate in stages 2 and 3 ookinetes and they also contain haemozoin pigment (Figure 3c and 3d). In stage 4 ookinetes, the protrusion reaches maximum length (Figure 3e). Stage 5 is the final stage of the maturation process. The mature ookinete is crescent shaped (sometimes club or fluke shaped) and has a distinct nucleus and haemozoin pigment (Figure 3f).

### Stage V ookinetes

**Figure 4:**
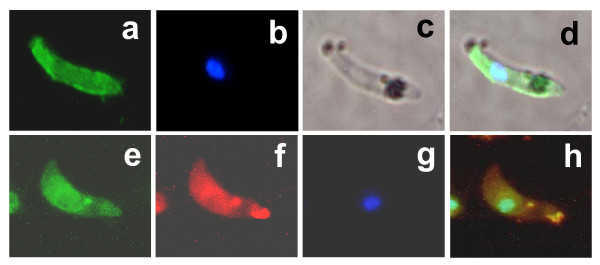
**Properties of ookinetes after culture in different media**. Upper panels: ookinete immuno-stained with anti-Pfs25 antibody. Fluorescent image due to antibody staining (a), fluorescent image of the nucleus due to DAPI staining (b), differential interference contrast (DIC) image (c) and merged image (d). Lower panels: double-labeling with anti-Pfs25 (e) and anti-Pf chitinase (f) antibodies. Nuclear staining with DAPI (g) and a merged image (h) are also shown. Picture shown here from transformation in ookinete medium E.

**Figure 5:**
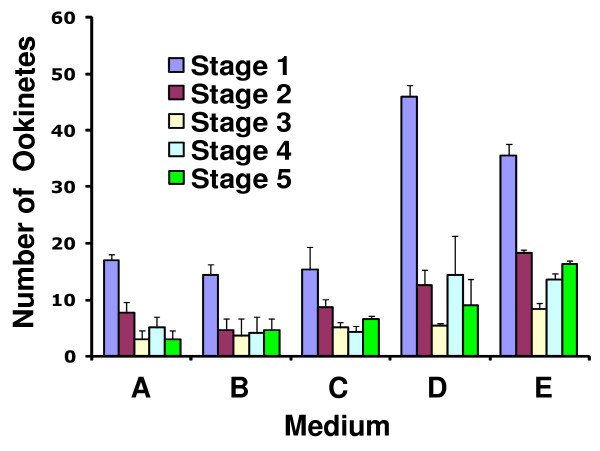
**Number of ookinetes per 10,000 RBCs at each stage of differentiation after 24 h of *vitro *culture in media A-E (Table 1)**. Gametocytes were washed, transferred to each of the five media and incubated at 24°C. The results shown here are from three independent experiments. Error bars are standard deviations.

Stage 5 ookinetes express Pfs25 on the surface and chitinase in the apical cytoplasmic region [12]. To maximize the identification accuracy of stage 5 mature ookinetes, only those parasites that were doubly-labeled by both anti-Pfs25 and anti-Pf chitinase antibodies (Figure 4) were counted. No significant differences were found in the number and proportion of ookinete developmental stages in media A-C (Figure 5). In media D and E, a higher proportion of stage 1 ookinetes were observed, presumably due to the presence of gametocyte activating factors. However, the higher stage 1 ookinete number in medium D did not result in more mature stage 5 ookinetes compared to medium E, implying that xanthurenic acid alone is not sufficient for the differentiation into fully mature stage V ookinetes. The number of stage 5 ookinetes in medium E - 15~18 ookinetes per 10,000 RBCs - was significantly higher than that for the other media, suggesting that other components in this medium promoted differentiation from stage 1 to stage 5. This represents a ~75-fold improvement over the original medium [4].

### Infectivity of stage V mature ookinetes

**Figure 6:**
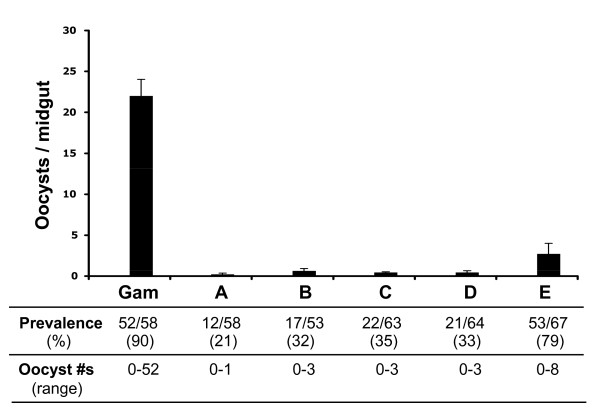
**Oocyst formation after feeding *An. gambiae *mosquitoes either a gametocyte culture or a 24-hour ookinete culture in either of the five media (A-E; Table 1)**. The graph shows mean oocyst numbers per mosquito midgut. Gam = Mosquitoes fed on gametocytes. Results are from three independent experiments. Error bars are standard deviations. Prevalence of infected mosquitoes and the range of oocyst numbers are shown at the bottom of the figure.

The ultimate test of ookinete functional viability is the measurement of the ability of ookinetes to develop into oocysts when fed to mosquitoes. When this test was applied to ookinetes produced in the five different ookinete media, it was observed that the oocysts number was very low in media A-D and that oocyst formation was highest - 2.5 oocysts per mosquito - with ookinetes produced in medium E (Figure 6). However, the number of oocysts formed after direct gametocyte feeding was about 10 times higher [13,14]. Prevalence (% infected mosquitoes) was similar for mosquitoes fed with ookinetes produced in media A to D and was much higher for mosquitoes fed with ookinetes produced in medium E (Figure 6).

This work sought to reproduce *in vitro *two essential steps of parasite development. The first occurs in the vertebrate host and involves differentiation of asexually dividing parasites into male and female gametocytes. The second occurs in the mosquito midgut and involves gametogenesis, fertilization and development into functional stage V ookinetes. This report focuses mostly on the second step. Modification of the existing procedures resulted in a dramatic improvement (about 75-fold) over the originally published procedure [4]. Key factors may include a slightly higher initial gametocyte yield, a slightly lower temperature (24 ± 2°C instead of 25-27°C) for the ookinete differentiation step and replacement of Fetal Bovine Serum by human serum in this study. Infectivity of *in vitro*-generated ookinetes to mosquitoes has not been measured previously. The experiments of Figures 5 and 6 illustrate the importance of this type of assessment: number of morphologically mature looking ookinetes is not a direct indication of success of mosquito infection. Factor(s) contained in the pupal extract and/or RBC lysate may be crucial for promoting functional viability.

## Conclusions

Taken together, the data presented in this study showed that use of human serum together with the combined addition of pupal extract and human RBC lysate in medium E enhances the full development of male and female gametocytes into mature ookinetes. Importantly, the *in vitro *differentiated ookinetes efficiently produced oocysts when fed to mosquitoes.

## Abbreviations

MR4: Malaria Research and Reference Reagent Resource Center; IgG: Immunoglobulin G; RBC: red blood cell; FBS: fetal bovine serum; DIC: differential interference contrast; PBS: phosphate buffered saline.

## Competing interests

The authors declare that they have no competing interests.

## Authors' contributions

AKG conceived the study, executed experiments and wrote a manuscript draft. RRD participated in the design of experiments and editing of figures. HI contributed with stained preparations and mosquito feeding. MJL participated in the design and coordination of the project and contributed to manuscript writing. All authors have read and approved the final manuscript.
